# Genetic Study of Four Candidate Holliday Junction Processing Proteins in the Thermophilic Crenarchaeon *Sulfolobus acidocaldarius*

**DOI:** 10.3390/ijms23020707

**Published:** 2022-01-09

**Authors:** Ryo Matsuda, Shoji Suzuki, Norio Kurosawa

**Affiliations:** Department of Environmental Engineering for Symbiosis, Graduate School of Science and Engineering, Soka University, 1-236 Tangi-machi, Hachioji, Tokyo 192-8577, Japan; ryomatsuda@soka.gr.jp (R.M.); s.suzukie@gmail.com (S.S.)

**Keywords:** hyperthermophilic archaea, *Sulfolobus acidocaldarius*, homologous recombination, Holliday junction, stalled replication fork

## Abstract

Homologous recombination (HR) is thought to be important for the repair of stalled replication forks in hyperthermophilic archaea. Previous biochemical studies identified two branch migration helicases (Hjm and PINA) and two Holliday junction (HJ) resolvases (Hjc and Hje) as HJ-processing proteins; however, due to the lack of genetic evidence, it is still unclear whether these proteins are actually involved in HR in vivo and how their functional relation is associated with the process. To address the above questions, we constructed *hjc*-, *hje*-, *hjm*-, and *pina* single-knockout strains and double-knockout strains of the thermophilic crenarchaeon *Sulfolobus acidocaldarius* and characterized the mutant phenotypes. Notably, we succeeded in isolating the *hjm*- and/or *pina*-deleted strains, suggesting that the functions of Hjm and PINA are not essential for cellular growth in this archaeon, as they were previously thought to be essential. Growth retardation in Δ*pina* was observed at low temperatures (cold sensitivity). When deletion of the HJ resolvase genes was combined, Δ*pina* Δ*hjc* and Δ*pina* Δ*hje* exhibited severe cold sensitivity. Δ*hjm* exhibited severe sensitivity to interstrand crosslinkers, suggesting that Hjm is involved in repairing stalled replication forks, as previously demonstrated in euryarchaea. Our findings suggest that the function of PINA and HJ resolvases is functionally related at lower temperatures to support robust cellular growth, and Hjm is important for the repair of stalled replication forks in vivo.

## 1. Introduction

Thermophiles inhabit a hot environment that accelerates the rate of DNA damage [1]. It has been proposed that DNA repair in thermophilic archaea has unique properties in order to be robust in extreme environments [2,3]. For example, no nucleotide excision repair function, which removes a broad spectrum of helix-distorting DNA lesions (such as UV-induced DNA damage, intrastrand crosslinks, and bulky adducts) has been identified in hyperthermophilic archaea; however, another DNA repair process has been proposed to have a homologous function [3,4,5]. An endonuclease XPF/Hef- and NucS/EndMS-deleted strain exhibits sensitivity to helix-distorting DNA lesions, suggesting that both endonucleases participate in homologous recombination (HR)-mediated stalled-fork DNA repair [3,4,5]. For these reasons, an opinion that this HR-mediated DNA repair serves as the major pathway for the removal of a wide variety of DNA lesions in hyperthermophilic archaea and is unusually efficient and reliable in comparison with other organisms has been argued [3,4,5]. Therefore, exploring the HR process in hyperthermophilic archaea is expected to expand our knowledge about the DNA repair mechanism in order to maintain genetic information.

HR is a fundamental mechanism that plays an important role in repairing stalled replication forks. The mechanism involves genetically exchanging homologous sequence processes to significantly enhance genome stability and genetic diversity. The process comprises four main steps. The first process is end resection catalyzed by the Rad50-Mre11-HerA-NurA complex producing 3′-single-stranded DNAs (ssDNAs). After end resection, ssDNA binding protein binds to 3′-ssDNA and protects the formation of the secondary structure of ssDNA [6,7]. In the second step, 3′-ssDNA is used for strand invasion, and the formation of four-stranded DNA is called the Holliday junction (HJ) catalyzed by recombinase RadA [8,9,10]. The third step is branch migration, in which the branch point of the HJ is moved by the activity of the branch migration helicases. In the final step, HJ resolvases bind to and cleave HJ [11,12], resulting in HJ resolution, and subsequent ligation ends the HR process. The role of putative branch migration helicases and HJ resolvases in HR in vivo and the functional relation of these proteins in vivo are not understood in detail (discussed in more detail below) [13,14,15,16].

To date, three helicases have been characterized as helicases with a branch migration activity (i.e., dissociation of a synthetic HJ to half junctions) in vitro in hyperthermophilic archaea. The orthologs of these proteins have also been found in most crenarchaeal and euryarchaeal species. Holliday junction migration (Hjm) from the hyperthermophilic euryarchaeon *Pyrococcus furiosus* was first discovered as a putative branch migration helicase with branch migration activity against a synthetic HJ [14]. In a previous study on the thermophilic crenarchaeon *Sulfurisphaera tokodaii* (formerly *Sulfolobus tokodaii*), Hjm has a fork regression activity against a chicken-foot structure known as the HJ in vitro [17]. Disruption of *hjm* led to lethality in the crenarchaeon “*Sulfolobus islandicus*” REY15A [18] (the double quotation marks indicate this species name has not been validated). In the hyperthermophilic euryarchaeon *Thermococcus kodakarensis*, the *hjm* knockout strain was constructed and was sensitive to UV irradiation and mitomycin C (MMC), respectively [4], suggesting that Hjm works at stalled replication forks to repair them. The second candidate is archaeal-long-helicase-related (Lhr1) [19,20]. Lhr1 from the euryarchaeon *Methanothermobacter thermautotrophicus* catalyzes branch migration of a synthetic HJ [20], and the thermophilic crenarchaeon *Sulfolobus acidocaldarius* Lhr1 (Saci_1500) unwinds a synthetic HJ, producing ssDNA [19]. However, the recombination frequency of the *lhr1*-deficient strain is identical to that of the parent strain [19], suggesting that the function of Lhr1 is not required for the HR process in vivo. As a third candidate, a recent study identified a novel helicase PilT N-terminal-(PIN)-domain-containing ATPase (PINA) from “*S. islandicus*” that promotes branch migration of a synthetic HJ in vitro [16]. PINA is likely to be responsible for branch migration in archaea, because it promotes branch migration, and its crystal structure is similar to RuvB, which is a branch migration helicase in bacteria [16]. A previous in vitro study proposed a putative role for Hjm and PINA in the regression of stalled replication fork, the formation of the chicken-foot structure, and HJ cleavage [21]. The branch-migration activity of the three candidates leads us to imagine that these helicases are involved in the repair of the stalled replication fork in vivo. However, it is unclear whether these candidates (especially Hjm and PINA) actually participate in the HR process in vivo because no genetic evidence has been provided.

HJ resolution is processed by two HJ resolvases, Hjc and Hje, in crenarchaeon. Hjc is conserved in all archaea, and Hje is a paralog of Hjc and is found in many species of the order *Sulfolobales* [12,22]. In thermophilic crenarchaeon, a biochemical study showed that the substrate specificity of Hjc and Hje are slightly different in *Saccharolobus solfataricus* (formerly *Sulfolobus solfataricus*), and a genetic study showed the *hje*-deficient strain of “*S. islandicus*” exhibited a high sensitivity to DNA-damaging agents HU, cisplatin, and MMS, which can cause replication fork-stalled and DNA double-strand breaks, while the *hjc*-deficient strain exhibited no sensitivity [22,23]. The cellular roles of Hjc and Hje seem to be different for HR-mediated DNA repair. However, similar to candidates for branch-migration helicase, it is still unclear whether both HJ resolvases are actually required for processive HR in vivo because no genetic evidence has been reported.

An in vitro study of Hjm and PINA has been reported; however, currently, *hjm*- and *pina*-deficient strains cannot be constructed in “*S. islandicus*”, and the sensitivity of mutants to DNA damage agents has not been examined [16,18]. In other words, the impact of Hjm and PINA on genome integrity has not been determined in detail. In addition, the relationship between Hje and these helicases is unclear [21]. The genetic study of two helicases (Hjm and PINA) and two nucleases (Hjc and Hje) will be useful for enhancing the understanding of the importance of branch migration and HJ resolution. In this study, we constructed *hjm*-, *pina*-, *hjc*-, and *hje*-knockout strains and double-knockout strains in *S. acidocaldarius* to investigate the functional role of Hjm and PINA in vivo, and the relationship between two helicases and two nucleases in archaeal HR.

## 2. Results

### 2.1. Construction of the hjc-, hje-, hjm-, and pina-Deleted Strains

**Figure 1:**
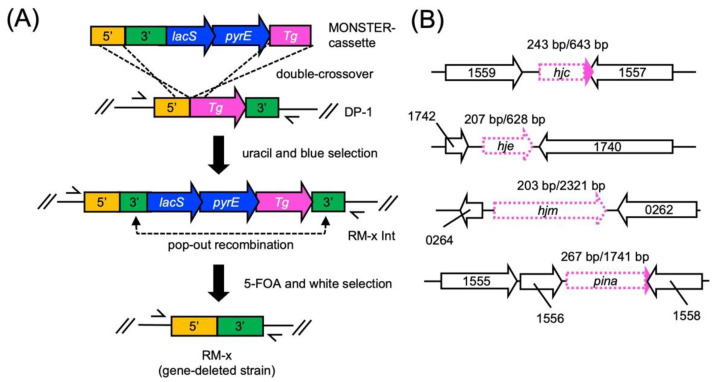
Construction of the gene-deleted strains using MONSTER. (**A**) Construction of genes (*hjm*, *pina*, *hjc*, and/or *hje*) encoding gene-deletion mutants. A plasmid-borne *pyrE-lacS* marker served as the PCR template, which attached *S. acidocaldarius* chromosomal sequences (5′, 3′, and partial sequences of target genes at the 5′ ends of the primers) to the ends of the selectable dual marker. After one-step construction, the MONSTER cassette was electroporated into strain DP-1. A double crossover between the MONSTER cassette and the chromosome at the 5′ and Tg regions results in the *pyrE-lacS* marker and 3′ region insertion at the target gene locus. The resulting uracil prototroph transformants that exhibit blue colonies can be selected on uracil-free plates. A target gene-deletion mutant with the marker removed was generated by pop-out recombination at two duplicated 3′ regions, which can be selected by 5-FOA counterselection in combination with X-gal staining. Arrows show the positions of the outer prime sets. (**B**) Setting deletion regions of the target genes. Magenta arrows indicate the target genes. The white region (dotted line) in magenta arrows indicates the locus of the deleted regions. The number above the target genes (magenta) indicates the length (bp) of the deleted sequence and whole sequence. The number in arrows indicates the gene number of Sac.

The gene-deleted strains were constructed by MONSTER as an unmarked-gene-deletion method (Figure 1) [24]. After transformation, 57 colonies/µg MONSTER-*hjc*, 31 colonies/µg MONSTER-*hje*, 18 colonies/µg MONSTER-*hjm*, and 71 colonies/µg MONSTER-*pina* grew under uracil selection. After blue visualization using X-gal solution, one blue colony was purified using single-colony isolation.

**Figure 2:**
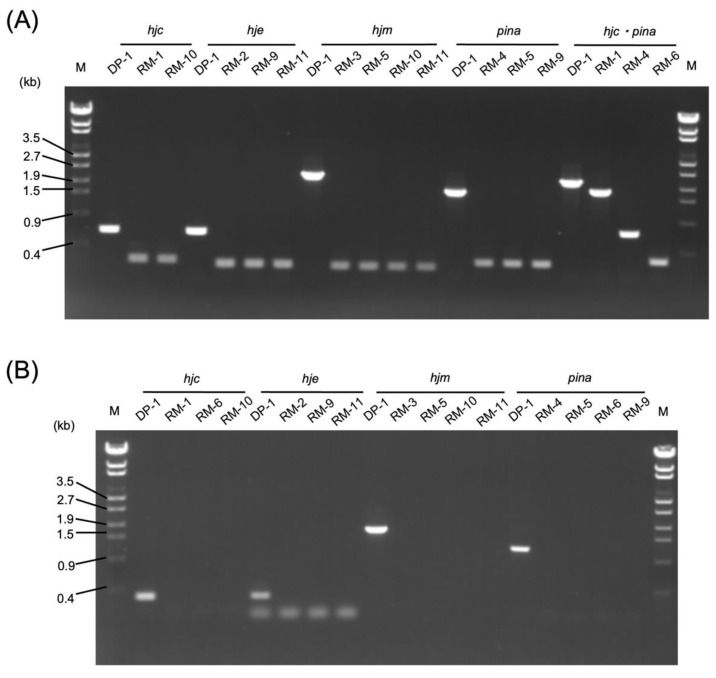
(**A**) PCR analysis of the *hjc*, *hje*, *hjm*, and *pina* of the locus of the gene-deleted strains using outer primers (Hjc-out-F/R, Hje-out-F/R, Hjm-out-F/R, PINA-out-F/R, and Hjc-out-F/PINA-out-F). The expected sizes of the PCR bands were as follows: 0.7 kb (DP-1), 0.2 kb (RM-1), and 0.2 kb (RM-10) in the *hjc* locus; 0.6 kb (DP-1), 0.2 kb (RM-2), 0.2 kb (RM-9), and 0.2 kb (RM-11) in the *hje* locus; 2.3 kb (DP-1), 0.2 kb (RM-3). 0.2 kb (RM-5), 0.2 kb (RM-10), and 0.2 kb (RM-11) in the *hjm* locus; and 1.7 kb (DP-1), 0.3 kb (RM-4), 0.3 kb (RM-5), and 0.3 kb (RM-9) in the *pina* locus; 2.4 kb (DP-1), 1.9 kb (RM-1), 1.0 kb (RM-4), and 0.2 kb (RM-6) in the *hjc* and *pina* locus. A λ-EcoT14 ladder was loaded in lane M. (**B**) PCR analysis of the *hjc*, *hje*, *hjm*, and *pina* of the locus of the gene-deleted strains using inner primers (Hjc-in-F/R, Hje-in-F/R, Hjm-in-F/R, and PINA-in-F/R). The expected sizes of the PCR bands were as follows: 0.3 kb (DP-1), no band (RM-1), no band (RM-6), and no band (RM-10) in the *hjc* locus; 0.3 kb (DP-1), no band (RM-2), no band (RM-9), and no band (RM-11) in the *hje* locus; 1.7 kb (DP-1), no band (RM-3), no band (RM-5), no band (RM-10), and no band (RM-11) in the *hjm* locus; and 1.2 kb (DP-1), no band (RM-4), no band (RM-5), no band (RM-6), and no band (RM-9) in the *pina* locus. A λ-EcoT14 ladder was loaded in lane M.

PCR analysis using outer primers confirmed that the blue colonies were intermediate transformants (named RM-1 Int, RM-2 Int, RM-3 Int, and RM-4 Int). A total of 2–3 × 10^8^ RM-x Int cells were spread on an XTUF plate for pop-out recombination. X-gal visualization revealed that 12, 8, 20, and 11 white colonies grew by plating RM-1 Int, RM-2 Int, RM-3 Int, and RM-4 Int cells, respectively. Five white colonies were randomly selected for the PCR analysis using outer primers. The genotypes of these colonies demonstrated the expected 0.3 kb, 0.5 kb, 2.1 kb, and 1.4 kb deletions in the *hjc*, *hje*, *hjm*, and *pina* loci, respectively (Figure 2A). In addition, PCR analyses using inner primers yielded no product from the gene-deleted strains (Figure 2B), indicating that the target genes were deleted from the original genomic locus and were not translocated on their genomes.

The gene-deleted strains were designated the *S. acidocaldarius* strains RM-1 (Δ*pyrE* Δ*suaI* Δ*phr* Δ*hjc*), RM-2 (Δ*pyrE* Δ*suaI* Δ*phr* Δ*hje*), RM-3 (Δ*pyrE* Δ*suaI* Δ*phr* Δ*hjm*), and RM-4 (Δ*pyrE* Δ*suaI* Δ*phr* Δ*pina*). In addition, double-knockout strains RM-5 (Δ*pyrE* Δ*suaI* Δ*phr* Δ*pina* Δ*hjm*), RM-6 (Δ*pyrE* Δ*suaI* Δ*phr* Δ*pina* Δ*hjc*), RM-9 (Δ*pyrE* Δ*suaI* Δ*phr* Δ*pina* Δ*hje*), RM-10 (Δ*pyrE* Δ*suaI* Δ*phr* Δ*hjm* Δ*hjc*), and RM-11 (Δ*pyrE* Δs*uaI* Δ*phr* Δ*hjm* Δ*hje*) were constructed. However, we could not construct the Δ*hjc* Δ*hje* (double-knockout) strain. Thus, the redundant function of Hjc and Hje in the HJ resolution and the essentiality of HJ resolution for cellular viability is evident in *S. acidocaldairus*. This result is compatible with a previous knockout study of the *hjc* and *hje* genes in “*S. islandicus*” [23].

### 2.2. Growth Characteristics of the Gene-Deleted Strains

**Figure 3:**
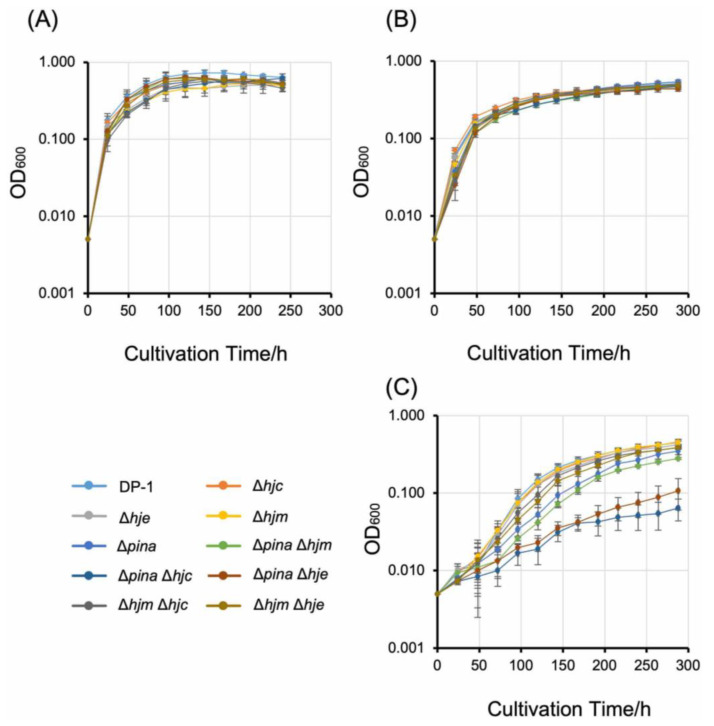
Growth curves of the gene-deleted strains. Overnight cultures of the gene-deleted strains and DP-1 strain were cultivated at 75 °C (**A**), 65 °C (**B**), and 55 °C (**C**). The error bars indicate ±SD calculated using three biological replicates.

The growth of deletion strains (RM-1 (Δ*hjc*), RM-2 (Δ*hje*), RM-3 (Δ*hjm*), RM-4 (Δ*pina*), RM-5 (Δ*pina* Δ*hjm*), RM-6 (Δ*pina* Δ*hjc*), RM-9 (Δ*pina* Δ*hje*), RM-10 (Δ*hjm* Δ*hjc*), and RM-11 (Δ*hjm* Δ*hje*)) and a parental strain DP-1 in the liquid culture was compared over a wide temperature range (55, 65, and 75 °C) (Figure 3).

The growth of all deficient strains was almost normal (final cell density; 0.54 ± 0.043–0.48 ± 0.043, the cultivation time for OD_600_ = 0.1; 15 ± 3.0 h–27 ± 6.8 h) at 75 °C in comparison with that of the parent strain (final cell density; 0.64 ± 0.011, the cultivation time for OD_600_ = 0.1; 14 ± 3.9 h) (Figure 3A). Similarly, at 65 °C, the growth of all deficient strains was also the same as that of the parent strain (Figure 3B). Similarly, at 55 °C, no marked difference was observed between the growth of Δ*hjc*, Δ*hje*, Δ*hjm*, Δ*hjm* Δ*hjc*, and Δ*hjm* Δ*hje*, and that of the parental strain (Figure 3C). In contrast, at 55 °C, the growth rate of Δ*pina* was lower than that of the parental strain (the cultivation time for OD_600_ = 0.1; 80 ± 11 h and 239 ± 84 h for DP-1 and Δ*pina*, respectively), but that of the final cell density remained almost normal (0.45 ± 0.023 and 0.35 ± 0.036 for DP-1 and Δ*pina*, respectively) (Figure 3C). The growth of Δ*pina* Δ*hjm* was similar to that of Δ*pina* (Figure 3C); notably, at 55 °C, the growth of Δ*pina* Δ*hjc* and Δ*pina* Δ*hje* was significantly retarded and was clearly lower than those of the parent strain and Δ*pina* (final cell density; 0.10 ± 0.020 and 0.11 ± 0.046 for Δ*pina* Δ*hjc* and Δ*pina* Δ*hje*, respectively; the cultivation time for OD_600_ = 0.1; 1013 ± 398 h and 704 ± 241 h for Δ*pina* Δ*hjc* and Δ*pina* Δ*hje*, respectively) (Figure 3C). This result demonstrated that none of the genes are solely essential for cellular growth under optimal growth temperatures; in contrast, Δ*pina* exhibited cold sensitivity, and double-knockout strains Δ*pina* Δ*hjc* and Δ*pina* Δ*hje* exhibited severe cold sensitivity in comparison with that of Δ*pina*.

### 2.3. Sensitivity of the Gene-Deleted Strains to Interstrand Crosslinker MMC

**Figure 4:**
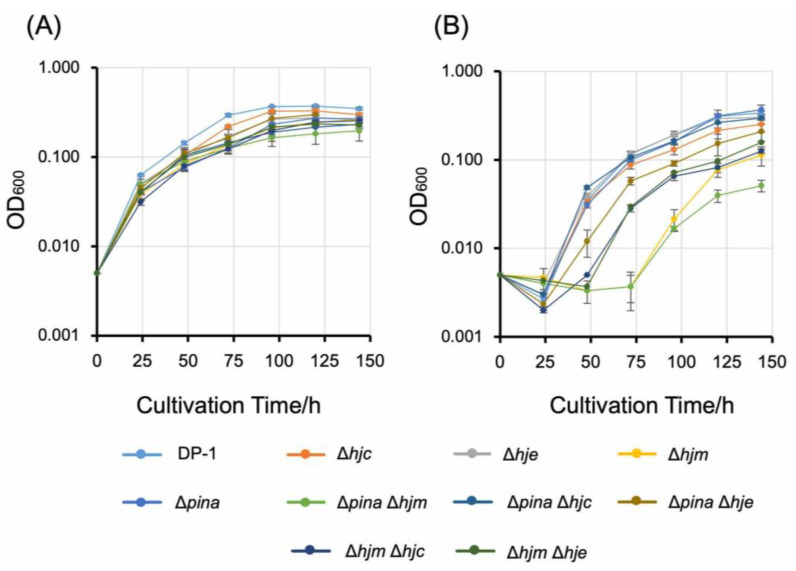
Growth curves of the knockout strains after MMC treatment. Mock treatment (**A**), MMC 240 µM (**B**). Error bars indicate ±SD calculated using three biological replicates.

We investigated the sensitivity of the gene-deleted strains to interstrand crosslinks induced by MMC by monitoring the growth in liquid culture at optimal growth temperatures after MMC treatment. Interstrand crosslinks cause severe damage to duplex DNA and stalled replication forks and strand breaks [25,26]. When mock treatment was performed, the growth of all gene deletion strains was similar to that of the parental strain (Figure 4A). After MMC treatment, a clear difference in growth between the gene-deleted strains and DP-1 was detected (Figure 4). Δ*hjc*, Δ*hje*, and Δ*pina* were not sensitive to MMC. In contrast, notably, Δ*hjm* exhibited a high sensitivity to MMC. The growth of Δ*pina* Δ*hje* became slightly lower after MMC treatment than that of DP-1 and Δ*pina*. These results show that Δ*hjm* has a high sensitivity compared to Δ*pina*.

## 3. Discussion

To explore the functional role and relationship of putative branch migration helicases (Hjm and PINA) and HJ endonucleases (Hjc and Hje) in the thermophilic crenarchaeon *S. acidocaldarius*, we constructed *hjc*-, *hje*-, *hjm*-, and *pina* single- and double-knockout strains and characterized the mutant phenotypes (growth characteristics and MMC sensitivity). Most disrupted strains were constructed, except for Δ*hjc* Δ*hje*. This exception is compatible with a previous genetic study in “*S. islandicus*” [19], suggesting that the function of Hjc and Hje is redundant and essential for cellular viability in *Sulfolobales* (discussed in more detail below). Interestingly, the *pina*-deleted strain (but not the *hjm*-deleted strain) exhibited growth retardation at a lower temperature (55 °C) (cold sensitivity), but not at a higher temperature (above 65 °C) (Figure 3A–C). This cold-sensitive phenotype was accelerated by additional deletion of the HJ resolvases Hjc or Hje (Figure 3C). These cold-sensitive phenotypes indicate that PINA is important for cellular growth at lower temperatures and that PINA and HJ endonucleases are functionally linked at lower temperatures in vivo. In addition, the *hjm*-deleted strain (but not the *pina*-deleted strain) exhibited sensitivity to the interstrand crosslinker MMC (Figure 4), indicating that Hjm is important for the DNA repair of interstrand crosslinks. This result is compatible with a previous genetic study in the euryarchaeon *T. kodakarensis* [3], suggesting that a function of Hjm in DNA repair is important among euryarchaea and crenarchaea.

Recent genetic studies point out that the HR process seems to be essential for hyperthermophilic archaea [3,4,16,18,27]. Our and previous knockout studies [23] demonstrated that the disruption of both Hjc and Hje may lead to lethality in crenarchaeon, suggesting that HJ is not resolved in the absence of either Hjc or Hje. In euryarchaeon Haloferax volcanii, double knockout of hjc and hef (not conserved in crenarchaea) led to lethality [28]. It is highly possible that Hjc, Hje, and Hef are responsible for the HJ resolvase function in this archaeon. Thus, HJ resolution is thought to be essential for cellular viability in both Sulfolobales and haloarchaeon [23,28]. Previous genetic studies reported that hjm and pina are essential for cellular viability in “S. islandicus” strain REY15A [16,18,29]. Notably, our results indicated that hjm and/or pina are not essential in S. acidocaldarius, suggesting that the functional roles of Hjm and PINA in cellular viability are different among the species/strains in the order Sulfolobales. In fact, hjm is not essential in the “S. islandicus” strain M.16.4 [30]. It is possible that another protein that complements the role of branch migration helicase is present in S. acidocaldarius but not conserved between the strains of “S. islandicus”. In addition, the reader should be aware that “Sulfolobus islandicu”’ is not a valid species (there is no paper describing its detailed morphological, physiological, biochemical, and chemotaxonomic characteristics) and is more closely related with Saccharolobus spp. rather than S. acidocaldarius. [31]. Even in euryarchaea, the essentiality of Hjm differs depending on the species. For example, Hjm (Hel308a) is essential in Haloferax vocalnii but not in T. kodakarensis [4,32]. Therefore, the importance of Hjm for cell survival is also different among the archaeal species. To elucidate the essentiality of hjm and pina in hyperthermophilic archaea, further knockout studies are needed.

Branch migration and HJ cleavages are performed by the relationship between helicase and nuclease. Previous biochemical studies have indicated that PINA enhances the cleavage activity of Hjc on a fixed HJ, and Hjm inhibits binding to HJ and the cleavage activity of Hjc [16,18]. It has also been reported that the branch-migration activity of PINA is suppressed by the binding of Hjc to HJ, and Hjm inhibits the binding of Hjc to HJ [16,18]. Although helicase can translocate along the molecule and separate base-paired regions, denaturation of the DNA at high temperatures supplemented helicase function. In our previous study, a single-strand binding protein (SSB) deleted strain of S. acidocaldarius grew well at a high temperature, but less grew at a lower temperature [33]. This suggested that the thermal destabilization of double-strand DNA (dsDNA) may complement the function of SSB at high temperatures, but not at lower temperatures. Taken together with previous findings and our results, it appears that an enhancement of HJ nuclease activity by the branch migration activity of PINA is especially required for normal growth at lower temperatures, but not optimal growth temperatures.

The pina-, hjc-, and hje knockout strains exhibited no sensitivity to the interstrand crosslinker MMC, indicating that repair of the stalled replication fork remains normal in the absence of PINA, Hjc, and Hje. On the other hand, Hjm is important for the DNA repair of interstrand crosslinks in S. acidocaldarius. In the euryarchaeon T. kodakarensis, disruption of hjm also led to a higher sensitivity to MMC [4]. Zhai et al. provided a model of the Hjm−PINA−Hjc interaction to repair stalled replication forks and HJ migration [21]. Taken together with our results, reversal of the replication fork by Hjm may be most important for the repair of stalled replication forks in both euryarchaea and crenarchaea [4,21]. However, Δhjm sensitivity was increased in comparison with those of Δhjm Δhjc and Δhjm Δhje after MMC treatment (Figure 4B). It is not possible to state clearly why this phenomenon was observed, but it was thought that the collapse of the stalled replication fork led to the removal of interstrand crosslinks in the absence of Hjm and HJ nucleases. Hef (not conserved in crenarchaea) and XPF are known as endonucleases that cleave stalled replication forks [3]. If Hjc and Hje inhibit the activity on the collapse of a stalled replication fork by an endonuclease (such as Hef and XPF), that will be the reason the sensitivity of Δhjm was higher than those of Δhjm Δhjc and Δhjm Δhje.

To date, the RecQ-like helicase Saci_1500, which unwinds HJ DNA in vitro, has been identified as another candidate putative branch migration helicase; however, the HR function in the Saci_1500 knockout strain is proficient [19]. It is interesting whether the RecQ-like helicase Saci_1500 does not participate in the HR process or whether other proteins (possibly Hjm and PINA) mask the HR-deficient phenotype in ΔSaci_1500. Further study to investigate the functional relation between Hjm, PINA, and the RecQ-like helicase Saci_1500 will be required.

## 4. Materials and Methods

### 4.1. Strains and Growth Conditions

The strains used in this study are listed in Table 1. The growth conditions were previously reported [24]. The *S. acidocaldarius* pyrimidine-auxotrophic and restriction endonuclease SuaI- and the DNA photolyase Phr-deficient strain DP-1 (Δ*pyrE* Δ*suaI* Δ*phr*) were used as the parent strains [24,34]. This strain and its derivatives were cultivated in xylose and tryptone (XT) medium (pH 3) [35] at 75 °C with or without shaking (160 rpm), as previously described [24]. For growth of the uracil (pyrimidine)-auxotrophic strain, 0.02 g/L uracil was added to XT medium (XTU). The XTU medium was supplemented with 50 μg/mL 5-FOA (XTUF) and used for counterselection with the pop-out recombination method. The solid plate medium was prepared as previously described [24].

### 4.2. General DNA Manipulation

The reagents used in these experiments were prepared as previously described [24].

### 4.3. Construction of Gene-Deleted Strains

The plasmids and DNAs used in this study are shown in Table 1, and the PCR primers used in this study are listed in Table 2. A multiple-gene-knockout system with one-step PCR (MONSTER) was used to prepare the *hjc* (Saci_1558), *hje* (Saci_1741), *hjm* (Saci_0263), and *pina* (Saci_1557) MONSTER cassettes (MONSTER-*hjc*, MONSTER-*hje*, MONSTER-*hjm*, and MONSTER-*pina*, respectively) and to construct the *hjc*-, *hje*-, *hjm*-, and *pina*-disrupted strains and double-knockout strains [24]. In brief, the MONSTER-*pina* cassette was amplified from placSpyrE as a template using MONSTER-*pina*-F/R primers (containing the 38-bp 5′ and 30-bp 3′ sequences of the *pina* flanking region and a 38-bp region of the *pina* as the target gene (Tg)-arm at the 5′ ends of the primers) and Emerald Amp MAX PCR Master mix (Takara Bio, Kusatsu, Shiga, Japan) under the following conditions: 94 °C for 3 min; 30 cycles of 94 °C for 30 s, 50 °C for 30 s, and 72 °C for 3 min; and a final extension at 72 °C for 3 min. Similarly, the other MONSTER cassettes, i.e., MONSTER-*hjc*, MONSTER-*hje*, and MONSTER-*hjm*, were amplified using the primers MONSTER-*hjc*-F/R, MONSTER-*hje*-F/R, and MONSTER-*hjm*-F/R. The purified PCR products (200 ng/μL in 5 mM Tris-HCl, pH 8.5) were used for the subsequent electrotransformation. Preparation of the electrocompetent cells and the transformation protocol were previously described in detail [24]. Gene deletion was performed using an optimal transformation protocol [24]. To disrupt the target gene, 2.0 μg of the MONSTER cassette was electroporated (15 kV/cm, 9 ms) into 200 μL of competent cells in a 2 mm electroporation cuvette (NEPA GENE, Ichikawa-shi, Chiba, Japan). Electroporation was performed using Gene Pulser II (Bio-Rad Hercules, CA, USA). After electroporation, 800 µL of MBS (modified Brock’s basal salt mixture) (pH 4.7) [24,36] was added and incubated for 30 min at 77–78 °C. The sample was spread onto an XT plate. After seven days of cultivation at 75 °C, the transformant colonies were stained blue by spraying a 10 mg/mL X-gal solution on the plate and incubating at 75 °C for one day. After the blue selection, several blue colonies were purified by single-colony isolation and analyzed by PCR screening using outer/inner primers (Table 2) that anneal with the outer/inner regions of target genes (deleted region).

### 4.4. Growth Temperature Range Test

To characterize the range of growth temperatures, overnight cultures (late-log to stationary phase) were inoculated into 6 mL of XTU liquid medium to yield an initial OD_600_ = 0.005. Inoculation was performed in triplicate using the same overnight culture. Cells were cultivated at 55, 65, and 75 °C without shaking on a block heater. Then, a cap of the test tube was loosely opened. The cell growth was monitored thereafter (Figure 3A–C).

### 4.5. Growth Curve after Treatment with DNA-Damaging Agent

For the mitomycin C (MMC) (Wako, Osaka, Japan) survival test, 200 µL of each overnight culture was collected by centrifugation and was resuspended in 100 µL of MMC in Milli-Q (zero and 240 µM). The cells were incubated in PCR tubes (ASTEC, Kasuya-gun, Fukuoka, Japan) at 75 °C for 2 h on a thermal cycler (GeneAtlas G, ASTEC). Then, the cells were harvested using centrifugation, washed once in 1 mL of 20 mM sucrose, and suspended in 200 µL of 20 mM sucrose. The diluted samples were inoculated with 6 mL of XTU liquid medium to yield an initial OD_600_ = 0.005. The cells were cultivated at 75 °C without shaking in an air incubator. Then, the cap of the test tube was closed. The cell growth was monitored thereafter.

## 5. Conclusions

To explore the functional role and relationship of putative branch migration helicases (Hjm and PINA) and HJ endonucleases (Hjc and Hje) in *S. acidocaldarius*, we constructed *hjc*-, *hje*-, *hjm*-, and *pina*-single- and double-deletion strains; examined the growth properties under high and low temperatures; and investigated the sensitivities of MMC. These results suggest that a function of Hjm and PINA is not essential for cellular growth in this archaeon. PINA is important for cellular growth at lower temperatures, and PINA and HJ endonucleases are functionally linked at lower temperatures. Hjm is important for the DNA repair of interstrand crosslinks, as previously demonstrated in euryarchaea. This study provides new insights into HR processes in thermophilic crenarchaeon.

## Figures and Tables

**Table 1:** Strains or DNA used in this study.

Strains or DNAs	Relevant Characteristic(s)	Source or Reference
**Strains**		
*S. acidocaldarius*		
DP-1	SK-1 with Δ*phr* (Δ*pyrE* Δ*suaI* Δ*phr*)	[24]
RM-1	DP-1 with Δ*hjc* (Δ*pyrE* Δ*suaI* Δ*phr* Δ*hjc*)	This study
RM-2	DP-1 with Δ*hje* (Δ*pyrE* Δ*suaI* Δ*phr* Δ*hje*)	This study
RM-3	DP-1 with Δ*hjm* (Δ*pyrE* Δ*suaI* Δ*phr* Δ*hjm*)	This study
RM-4	DP-1 with Δ*pina* (Δ*pyrE* Δ*suaI* Δ*phr* Δ*pina*)	This study
RM-5	RM-4 with Δ*hjm* (Δ*pyrE* Δ*suaI* Δ*phr* Δ*pina* Δ*hjm*)	This study
RM-6	RM-4 with Δ*hjc* (Δ*pyrE* Δ*suaI* Δ*phr* Δ*pina* Δ*hjc*)	This study
RM-9	RM-4 withΔ*hje* (Δ*pyrE* Δ*suaI* Δ*phr* Δ*pina* Δ*hje*)	This study
RM-10	RM-3 with Δ*hjc* (Δ*pyrE* Δ*suaI* Δ*phr* Δ*hjm* Δ*hjc*)	This study
RM-11	RM-3 with Δ*hje* (Δ*pyrE* Δ*suaI* Δ*phr* Δ*hjm* Δ*hje*)	This study
**Plasmid DNA**		
placSpyrE	Plasmid DNA carrying 0.8 kb of 5′ and 3′ homologous regions of *suaI* locus at both ends of *pyrE*-*lacS* dual marker	[24]
**PCR products**		
MONSTER-*hjc*	Linear DNA containing the 38-bp 5′ and 30-bp 3′ sequences of the *hjc* flanking regions, and a 38-bp region of *hjc* as the Tg-arm at both ends of *pyrE*-*lacS* dual marker, respectively	This study
MONSTER-*hje*	Linear DNA containing the 38-bp 5′ and 30-bp 3′ sequences of the *hje* flanking regions, and a 38-bp region of *hje* as the Tg-arm at both ends of *pyrE*-*lacS* dual marker, respectively	This study
MONSTER-*hjm*	Linear DNA containing the 38-bp 5′ and 30-bp 3′ sequences of the *hjm* flanking regions, and a 38-bp region of *hjm* as the Tg-arm at both ends of *pyrE*-*lacS* dual marker, respectively	This study
MONSTER-*pina*	Linear DNA containing the 38-bp 5′ and 30-bp 3′ sequences of the *pina* flanking regions, and a 38-bp region of *pina* as the Tg-arm at both ends of *pyrE*-*lacS* dual marker, respectively	This study

**Table 2:** Primers used in this study.

Primers	Sequence (5′-3′) *
MONSTER-*hjc*-F	ttagagatataattctgagaggaaaacctaaatcctaa**gtaaggtatgttgagggaaaaataagtaaa**TGTTTTTCTCTATATCAATCTC
MONSTER-*hjc*-R	cctttagttttattactcacataataaaataaaaacacACTCCTAGATCTAAAACTAAAG
MONSTER-*hje*-F	cactcctttttaaggcttatcagacaattttggtgcaa**tatttatttttcctgttagcgagatgtaag**TGTTTTTCTCTATATCAATCTC
MONSTER-*hje*-R	agttccctttcagcactttttccaatgtctctattcatACTCCTAGATCTAAAACTAAAG
MONSTER-*hjm*-F	cttataaatcctacaaaataatgggtaacgtttaggat**ttacttaaacggtaaagtgacatttaagga**TGTTTTTCTCTATATCAATCTC
MONSTER-*hjm*-R	ctatcgacaggtaaatcttctacggtaatttcttccatACTCCTAGATCTAAAACTAAAG
MONSTER-*pina*-F	gtatgaaataaaatcctattcagaagggtattttagtc**tatgagaaaaagtacaagataaagataaga**TGTTTTTCTCTATATCAATCTC
MONSTER-*pina*-R	agagcagacttatctggaagtaaatctcttgcaggcaaACTCCTAGATCTAAAACTAAAG
Hjc-out-F	gcaaatactatcaaagaagg
Hjc-out-R	tgtttaataaaaaagttgtctc
Hje-out-F	taggaagcaaataaatctatc
Hje-out-R	aaagagttaggaactcattg
Hjm-out-F	aaaggaaaagcttattaatgg
Hjm-out-R	tctatacgacttttcttacc
PINA-out-F	ttcatcctgaattatcagag
PINA-out-R	attatgttgcggatttagag
Hjc-in-F	tgagagatatcttgtttcaag
Hjc-in-R	tgacttaattgtctctaaatcc
Hje-in-F	ctgtggtatgtagttctctagg
Hje-in-R	cctagagaactacataccacag
Hjm-in-F	ttatggcagaattaggtatg
Hjm-in-R	actctgaccaactctaacaac
PINA-in-F	tttcgaagtacattgagaac
PINA-in-R	ttctccaaaaacgtatatctc

* A common sequence for amplification of the *pyrE*-*lacS* dual marker, and 5′, 3′, and Tg regions were indicated by capital letter, underline, bold, and double line, respectively.

## Data Availability

The original contributions presented in the study are included in the article, and further inquiries can be directed to the corresponding author.
